# Rat Fall Surveillance Coupled with Vector Control and Community Education as a Plague Prevention Strategy in the West Nile Region, Uganda

**DOI:** 10.4269/ajtmh.17-0502

**Published:** 2017-10-23

**Authors:** Karen A. Boegler, Linda A. Atiku, Russell E. Enscore, Titus Apangu, Joseph Tendo Mpanga, Sarah Acayo, John Kaggwa, Paul S. Mead, Brook M. Yockey, Kiersten J. Kugeler, Martin E. Schriefer, Kalanthe Horiuchi, Kenneth L. Gage, Rebecca J. Eisen

**Affiliations:** 1 Centers for Disease Control and Prevention, Division of Vector-Borne Diseases, Fort Collins, Colorado;; 2 Uganda Virus Research Institute, Entebbe, Uganda

## Abstract

Plague, primarily a disease of rodents, is most frequently transmitted by fleas and causes potentially fatal infections in humans. In Uganda, plague is endemic to the West Nile region. Primary prevention for plague includes control of rodent hosts or flea vectors, but targeting these efforts is difficult given the sporadic nature of plague epizootics in the region and limited resource availability. Here, we present a community-based strategy to detect and report rodent deaths (rat fall), an early sign of epizootics. Laboratory testing of rodent carcasses is used to trigger primary and secondary prevention measures: indoor residual spraying (IRS) and community-based plague education, respectively. During the first 3 years of the program, individuals from 142 villages reported 580 small mammal deaths; 24 of these tested presumptive positive for *Yersinia pestis* by fluorescence microscopy. In response, for each of the 17 affected communities, village-wide IRS was conducted to control rodent-associated fleas within homes, and community sensitization was conducted to raise awareness of plague signs and prevention strategies. No additional presumptive *Y. pestis*-positive carcasses were detected in these villages within the 2-month expected duration of residual activity for the insecticide used in IRS. Despite comparatively high historic case counts, no human plague cases were reported from villages participating in the surveillance program; five cases were reported from elsewhere in the districts. We evaluate community participation and timeliness of response, report the frequency of human plague cases in participating and surrounding villages, and evaluate whether a program such as this could provide a sustainable model for plague prevention in endemic areas.

Plague is a life-threatening, flea-borne, rodent-associated zoonosis caused by *Yersinia pestis*. The plague bacterium has a nearly global distribution; however, in recent decades most plague cases have been reported from East Africa and Madagascar.^1,2^ In Uganda, plague is endemic in the highlands of the far northwest, which are known as the West Nile region.^3–6^ Here, *Y. pestis* is maintained in enzootic cycles among sylvatic and peridomestic rodents and their fleas, with *Arvicanthis niloticus* and *Crocidura* sp. likely playing important roles in plague epizootics.^7^ During plague epizootics, infections are assumed to spill over into *Rattus rattus*, which is commonly encountered within households in the West Nile region, is highly susceptible to plague infection, and harbors efficient *Y. pestis* vectors (*Xenopsylla cheopis* and *Xenopsylla brasiliensis*). Based on these findings, humans are presumed to be exposed to plague bacteria most commonly in and around homes when rats die, forcing their potentially infectious fleas to find an alternative host, including humans.^6,8^ Most plague cases occur between the months of September and December in the West Nile region.^4,9^ However, the number of cases occurring per year is highly variable; interannual variation in suspect plague cases has been correlated with seasonal rainfall patterns.^9^

Primary prevention of human plague typically focuses on vector control or rodent reduction within limited areas affected by plague epizootics.^10^ Recent studies from the West Nile region have shown that indoor residual spraying (IRS) and insecticide delivery tubes effectively reduce flea loads on rodents in the home environment where most exposures are believed to occur.^6,11,12^ While most households in a 2013 study self-reported using some form of rodent control, such as lethal trapping, application of rodenticide, or cat ownership;^13^ thus far, rodent control, specifically the use of poisons, or traps, have not proven to be effective given the massive resources required to sustain suppression of populations.^14^ Secondary prevention of plague aims to reduce case fatality rates through education campaigns that emphasize recognition of signs of plague and urging persons with symptoms consistent with plague to seek care without delay.^10^ Early diagnosis followed by an appropriate antibiotic therapy significantly improves outcomes of patients with plague.^15^

Although the factors that trigger epizootics remain poorly defined,^16^ previous environmental investigations of human plague cases in the West Nile region have noted that villagers commonly report seeing a larger than usual number of rat carcasses (referred to as a “rat fall”) before the onset of human plague cases.^4,5,7^ We wanted to know if we could capitalize on these observations, using them to inform timely, targeted interventions to prevent human cases.

Here, we describe an animal-based surveillance and early response program (herein referred to as rat fall surveillance or RFS) that engages members of the community, volunteer village health teams (VHTs), subcounty environmental health officers, and local leaders. Through the program, rat falls are reported, carcasses are collected and tested, and *Y. pestis*-positive results trigger community education and target implementation of vector control (IRS) to prevent human plague cases. Our specific objectives are to evaluate community participation and timelines of response under the RFS program, and to report frequency of human plague cases in participating and surrounding villages. More broadly, we aim to describe the success and limitations of this community-based plague prevention program and evaluate whether RFS might be applied successfully within resource-limited endemic areas.

## MATERIALS AND METHODS

### Program location and village participation.

**Figure 1.**
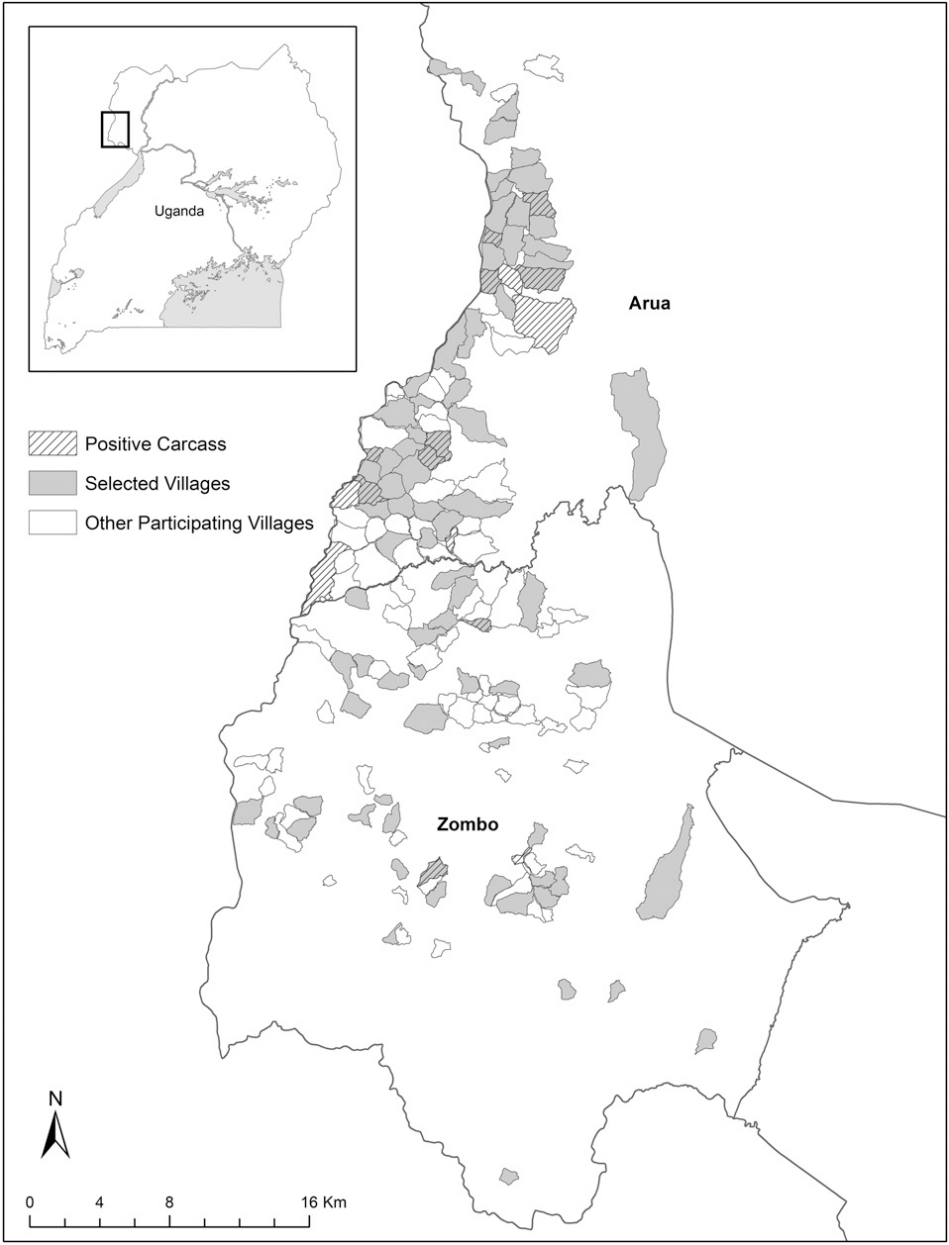
Map showing 83 villages selected for participation in the rat fall surveillance program, other “out of network” villages that also submitted carcasses and villages that submitted a *Yersinia pestis*-positive carcass. Inset: The program area of interest, in the West Nile region of Uganda.

This program was implemented and evaluated in the Arua and Zombo districts of the West Nile region, Uganda, from July 1, 2013 to June 30, 2016. A total of 83 villages, representing a local estimate of approximately 37,000 persons, were selected for participation among 563 local villages with a history of plague and included many of those reporting the greatest number of confirmed or suspect human plague cases between 1999 and 2011 (Centers for Disease Control and Prevention, unpublished data) (Figure 1). To expand the geographic extent of the surveillance network, in instances where neighboring villages reported confirmed cases and high case counts, a village may have been excluded so that a geographically distant village could be included, even if the more distant village had lower case counts. Seventy-five villages were invited to participate in the program between July and September of 2013, and an additional six villages, which were described in a concurrent study^6^ were added to the surveillance network in September 2014. During the evaluation period, two villages were elected to split into four; these four villages were treated separately during analysis.

The 83 selected villages were typical of those situated throughout the two districts where subsistence farming is the dominant occupation type, and most families live in huts of traditional construction (having mud walls and grass-thatched roofs).^13^ Within each village, one VHT member, a volunteer elected by local leaders and under the direction of subcounty environmental health officers to support the goals of the Uganda Ministry of Health, was invited to participate in the program.

### Identification of rat falls, carcass testing, and response.

**Figure 2.**
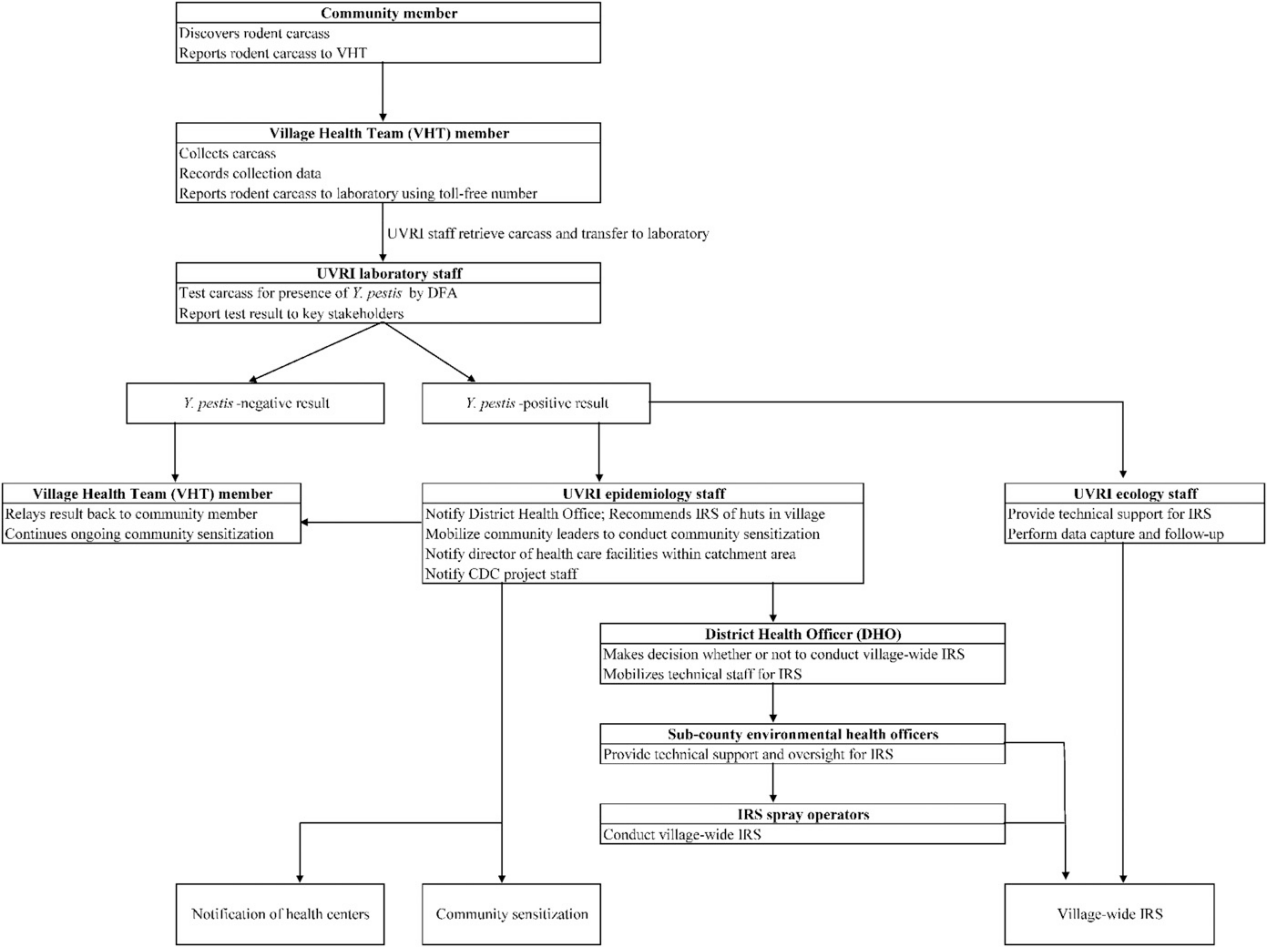
Schematic overview of critical communication and action steps for the rat fall surveillance and response program. Final response steps include notification of the village health teams (VHT) (for *Yersinia pestis*-negative carcasses), community sensitization, and indoor residual spraying (IRS) (for *Y. pestis*-positive carcasses).

A schematic overview of the RFS and response program is shown in Figure 2. As part of the surveillance program, VHTs investigated reports of dead rodents from village residents, collected any carcasses found using basic universal precautions, and notified Uganda Virus Research Institute (UVRI) staff by cell phone call to a preprogrammed toll-free phone number of the need for sample transfer and testing. Particularly because rodenticide use and other methods of rodent control are not uncommon in the villages, for the purposes of the program; a “rat fall” was defined as the discovery of one or more small mammal carcasses in the absence of rodenticide use or obvious injury. At the time of carcass collection, VHTs recorded date and location information and searched an area at least 100 m in all directions to locate any additional carcasses, inquiring with neighboring households to determine if any other carcasses had been found. Carcasses were stored at an ambient temperature until transferred to the UVRI staff for transport to the laboratory for testing.

Processing, testing, and storage of potentially infectious carcasses were conducted in an access-controlled laboratory under BSL-2 conditions. When possible, carcasses were identified to species using a published key.^17^ Carcasses were then necropsied and touch-preparation slides of the liver and spleen tissues were tested for the presence of *Y. pestis* F1 antigen using a direct fluorescent antibody (DFA) assay described elsewhere.^18^ Carcasses were considered presumptive positive for *Y. pestis* and actionable if the presence of the *Y. pestis* F1 antigen was detected in one or both tissues. For the purpose of brevity, we later refer to carcasses testing positive or equivocal by DFA as *Y. pestis*-positive.

When a carcass tested positive for *Y. pestis*, UVRI staff notified the District Health Officer, Uganda Ministry of Health to recommend timely IRS treatment of all the huts in the reporting village to reduce the numbers of hut-associated, potentially infectious fleas. For the duration of the program reported here, a wettable powder formulation of bendiocarb (Ficam-VC, 800 g/kg, Bayer, Ltd. Isando, South Africa) was applied to interior walls and floors after a modified application method described previously.^12^ The residual activity of Ficam-VC against rodent-associated fleas was unknown; therefore, the minimum expected duration of activity against malaria vectors on mud surfaces, or 2 months, was assumed.^19,20^

In addition to IRS, a number of community sensitization efforts were initiated in response to *Y. pestis*-positive carcass results. Immediately after notification of the test result, the VHT reported the finding directly to the household where the carcass was found. UVRI staff, together with village leadership, hosted meetings at places of worship, schools, markets, and other community spaces to raise awareness of plague at the village level and to share messages of primary and secondary plague prevention. Finally, the UVRI staff notified the health care facilities within the catchment area of the affected village, as well as local traditional healers to alert them to the increased potential for human plague cases.

When *Y. pestis* was not detected in a carcass, the laboratory staff directly notified the submitting VHT who then gave the result to the individual(s) who reported the carcass. After notification of the person(s) reporting the small mammal death, no further response actions were initiated.

### Participant feedback and program improvement.

To improve the surveillance and response program and address specific programmatic issues, UVRI and Centers for Disease Control and Prevention (CDC) leadership elicited feedback from VHTs, district- and sub-county-level representatives, environmental health officers, field and laboratory staff, and others annually at refresher training workshops and through organized periodic meetings with key stakeholders. To identify where disposable supplies were needed, what equipment types were useful or problematic, and which areas of the communication or response program could be improved, the UVRI staff also contacted VHTs by phone on a monthly basis and responses were summarized.

### Data analysis.

Village-level reporting data, notification, and test result dates were used to evaluate the timeliness of the RFS program for both *Y. pestis*-positive and *Y. pestis*-negative carcasses. The overall metric used to evaluate the timeliness of the program was the mean or median number of days between the initial report of a small mammal carcass and the completion of the response.

Statistical comparisons of various response times were made for all carcasses between years 1, 2, and 3 using analysis of variance among them, assuming unequal variance, and if any statistically significant difference was found; a Tukey’s multiple comparison of means test was used to identify where the difference lay. Difference of medians were compared using the Wilcoxon–Mann–Whitney exact test. The Mann–Kendall test was used to evaluate whether a trend existed in IRS response times, whereas a linear model with periodic covariates was used to evaluate carcass submissions by month.

Proportions were compared using a mid-p exact test for two proportions and a Fisher’s exact test for three proportions. For all statistical comparisons, significance was declared at the alpha = 0.05 level. Data summarization was performed using the JMP software suite,^21^ whereas figures and comparisons were produced using R statistical software.^22^

### Ethics determinations.

Before the initiation of the program, all protocols were reviewed and approved by the UVRI Research Ethics Committee, Uganda National Council for Science and Technology, and the Uganda President’s Office. Review by the CDC, the National Center for Emerging and Zoonotic Infectious Diseases Human Subjects Coordinator determined the protocols for this program to be nonhuman subjects’ research, thus a full committee review was not required.

## RESULTS

### Participant involvement and program acceptability.

Of the 83 participating VHTs, 68 (80%) reported and submitted at least one small mammal carcass through the program during the 3 years of evaluation. In addition, small mammal deaths were reported through spontaneous channels of communication by community members from another 74 villages throughout the region that were not initially selected for participation in the program (Figure 1). During brief follow-up interviews, those who submitted carcasses for testing from these “out of network” villages reported hearing about RFS through community sensitization efforts or through work or personal contacts. Overall, more carcasses were submitted from participating villages than “out of network” villages (432 versus 148). Continued use of the RFS program, defined as the submission of more than one carcass over 3 years, was observed from participating villages (58 of 68, or 85.3%) as well as “out of network” villages (37 of 74, or 50.0%).

### Summary of submissions.

**Figure 3.**
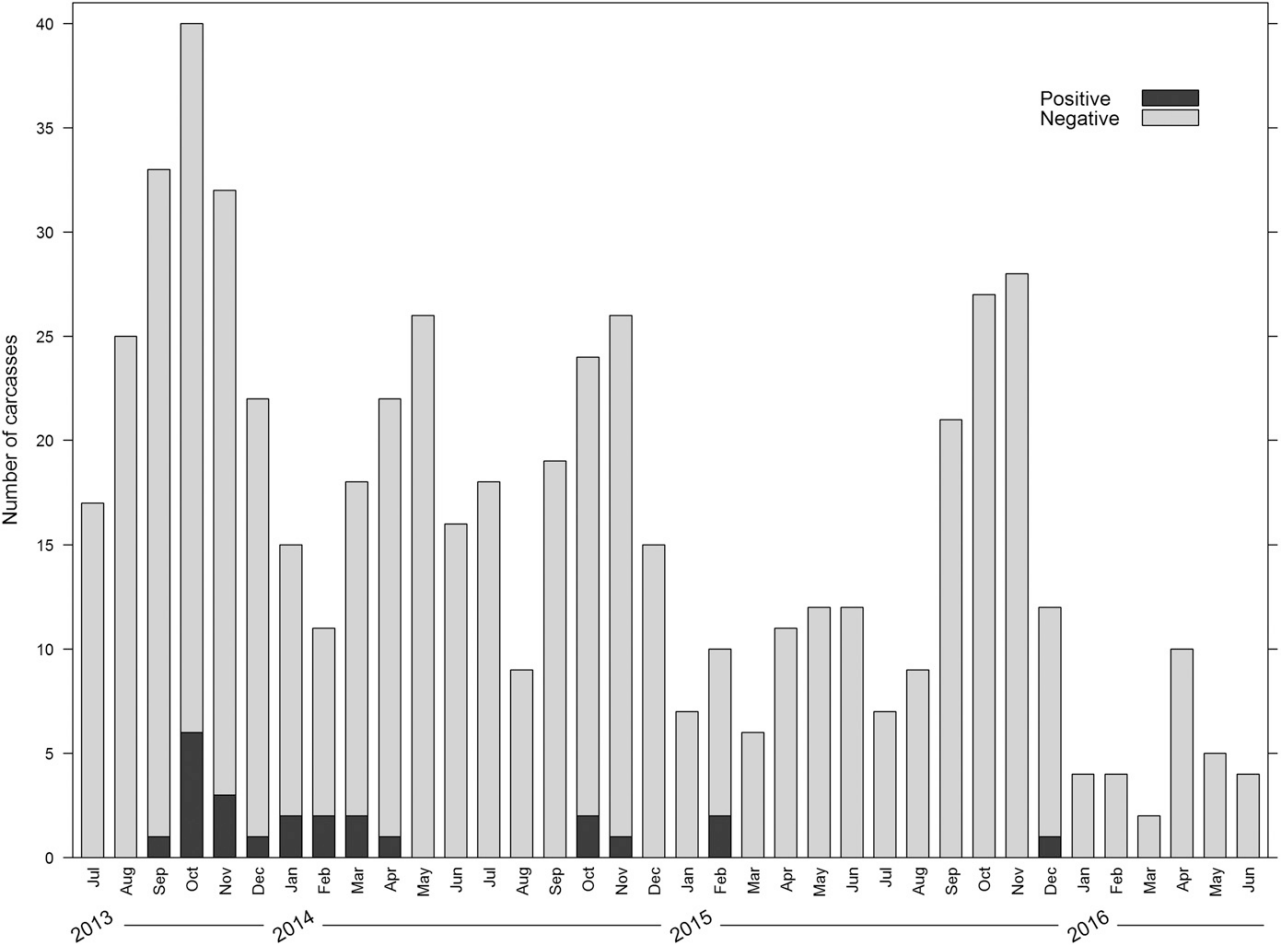
Distribution of *Yersinia pestis*-positive and negative small mammal carcasses submitted through the rat fall surveillance program by month between July 1, 2013 and June 30, 2016.

Between July 1, 2013 and June 30, 2016, a total of 580 small mammal carcasses were submitted through the RFS program. Of the 523 calls received over the 3-year surveillance period, most (*N* = 484; 92.5%) were made to report the observation of a single carcass, whereas the remainder (*N* = 39; 7.5%) were made to report multiple carcasses on a single day. Small mammal deaths were reported to the surveillance program in every month of the year (Figure 3), with a median of 15 carcasses submitted per month (range: 2–40, mean: 16.1) and a statistically significant decreasing trend over time (*P* < 0.0001). The most commonly reported species was *R. rattus* (*N* = 432), which represented 74.6% of the carcasses submitted, followed by *A. niloticus* (*N* = 90; 15.5%), and *Mastomys* sp. (*N* = 16; 2.8%); the remaining nine other species combined (*N* = 27) represented 4.7% of total submissions. A small number of carcasses were not identifiable because of poor condition of the carcass (*N* = 14; 2.4%). Species belonging to the genera *Mastomys* and *Crocidura* are difficult to distinguish morphologically; therefore, these identifications were made at the genus level. Based on earlier molecular identifications from the same region, however, small mammals identified as *Mastomys* spp. were likely either *Mastomys natalensis* or *Mastomys erythroleucus* and *Crocidura* spp. were most likely *Crocidura olivieri*.^7^ Of the carcasses for which location of collection data were available (*N* = 567), 69.1% were found indoors (*N* = 392), 30.5% were found near the home (*N* = 173) and 0.4% were found more than 30 m from the home (*N* = 2).

### Laboratory testing of carcasses.

Of 580 carcasses submitted, 24 (4.1%) tested positive by DFA for *Y. pestis*, 555 carcasses (95.7%) tested negative for *Y. pestis*, and 1 (< 1%) was too desiccated to test. All carcasses that tested *Y. pestis*-positive were reported between the months of September and April (Figure 3). Most carcasses testing *Y. pestis*-positive were *R. rattus* (19, 79.2%), whereas *A. niloticus* and *Mastomys* sp. comprised 16.7% and 4.2% of the total, respectively. There was not a statistically significant difference in infection prevalence among *R. rattus* (4.4% positive of 433 submitted), *A. niloticus* (4.4% of 90), and *Mastomys* sp. (6.3% of 16) (*P* = 0.72). Although less commonly reported, small mammal carcasses submitted in groups (> 1 per day from the same village), were significantly more likely to test positive for *Y. pestis* (10.3% of 39 carcass groups) than carcasses submitted singly (2.5% of 484 carcasses) (Difference: 7.8%, 95% confidence interval [CI]: 1.3–21.2%, *P* = 0.03). There was no significant difference in the percent of carcasses testing positive for *Y. pestis* submitted from participating and “out of network” villages (3.3% and 5.4% respectively, difference: −2.1%, 95% CI: −6.6–2.4%, *P* = 0.26).

### Intervention efforts in response to positive carcasses.

**Figure 4.**
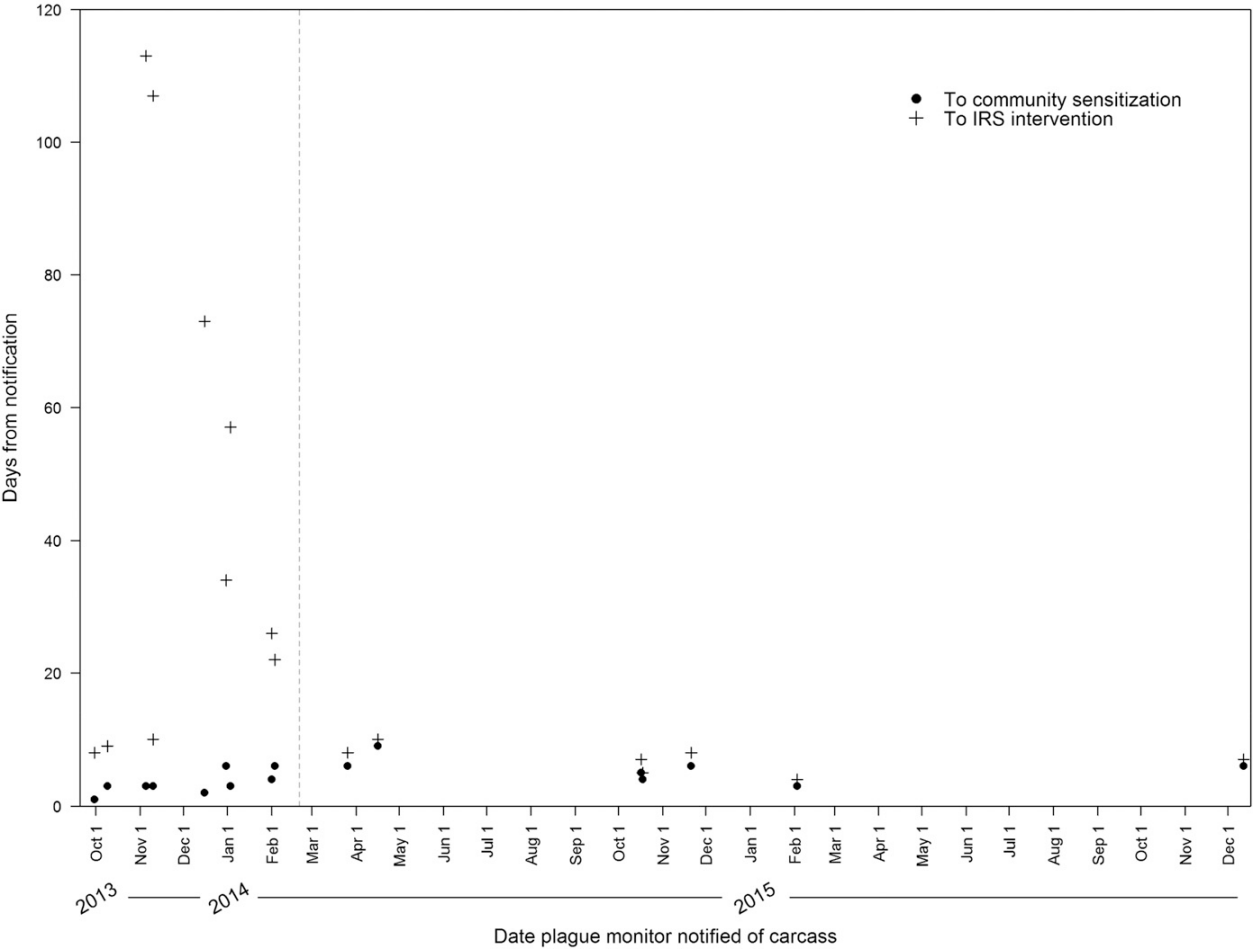
Number of days after initial report of positive carcass until village-wide indoor residual spraying (IRS) completed. Dashed line indicates the date of key stakeholder meeting.

During the 3 years of program evaluation, 17 village-wide IRS applications were enacted in response to the submission of at least one *Y. pestis*-positive carcass, including one village that was treated twice, once in November 2013 and again in November 2014 (Figure 4). Ten sprays were conducted in participating villages, whereas seven were conducted in “out of network” villages. The number of family huts per village ranged from 112 to 544, with a median village size of 230 huts. Hut-level IRS coverage in these villages ranged from 52% to 100%, with a median of 93.5%. Reasons for nontreatment of targeted huts included inaccessibility (locked huts on the date of spray), occupancy by sick residents or newborn babies, or when householders did not have a suitable place to temporarily store their possessions.

In each of the 17 affected villages, community-wide meetings were held to communicate messages about plague prevention, the signs and symptoms of plague infection, and the importance of early treatment. These meetings were held in community spaces, including places of worship, schools, and markets, and attendees included residents and local leaders from the affected village and surrounding areas.

### Timeliness of reporting, testing, and response efforts.

The average number of days between critical communication and action steps are shown in Table 1. For all small mammal carcasses submitted during the 3 years of program evaluation, the average time elapsed from notification to response (including the report from the community, carcass collection and transfer to the laboratory, DFA testing, and notification of the test result to the VHT) was fewer than 2 days. When this same metric was evaluated separately for each year of the program, average time to response for all carcasses showed a statistically significant decrease from year 1 to year 3 (Table 1). The timeliness of specific action steps, including retrieval and submission of carcasses to the laboratory and the communication of test results, likewise improved significantly over time (Table 1). Overall time from community report of a carcass to VHT being notified of the test result was similar for both *Y. pestis*-positive and negative carcasses (data not shown). For all comparisons, the final report of test results back to the community member(s) who observed the carcass was conducted by the VHTs; dates of this final response step were not recorded and could therefore not be evaluated.

**Table 1:** Mean (95% confidence interval) time in days between each critical step in the surveillance and reporting program for all carcasses submitted between July 1, 2013 and June 30, 2016

Year(s) of program	Community reports carcass. VHT investigates and collects carcass	Carcass reported by phone, then retrieved and submitted to laboratory for testing	Laboratory testing	Communication of test result back to VHT	Community member reports carcass and VHT is notified of test result
Year 1	0.31 (0.24–0.39)	0.34 (0.26–0.43)	0.92 (0.75–1.08)	0.57 (0.37–0.78)^a,b^	2.04 (1.76–2.31)^d^
Year 2	0.30 (0.21–0.40)	0.26 (0.17–0.35)	0.93 (0.83–1.04)	0.22 (0.14–0.30)^a,c^	1.70 (1.53–1.88)
Year 3	0.33 (0.23–0.44)	0.17 (0.04–0.29)	0.89 (0.75–1.03)	0.07 (0.02–0.13)^b,c^	1.44 (1.29–1.60)^d^
Years 1–3*	0.27 (0–0.69)	0.41 (0.19–0.63)	0.83 (0.31–1.36)	0.29 (0.10–0.49)	1.83 (1.25–2.41)

VHT = village health teams. Statistical comparisons between the first, second, and third years of the program are shown; significant differences between means are indicated by matching superscript characters. Right-hand column shows total time between first and last steps of the submission and result communication chain.

* Summary data for all years combined was not included in statistical comparisons.

The number of days between the detection of the first *Y. pestis*-positive carcass from a village and completion of the two main response efforts, IRS and community sensitization, are shown in Figure 4. For the 17 village-wide interventions, IRS was completed within a median of 10 days (range: 4–113) and mean of 29.9 days (95% CI: 11.5–48.3) after the initial report of a carcass by community members, and village-level community sensitization efforts were conducted within a median of 4 days (range: 1–9) and mean of 4.3 days (95% CI: 3.3–5.3). The timeliness of community sensitization efforts had a statistically significant increase across the 3 years of the study (*P* = 0.01).

Intermediate communication steps after the detection of *Y. pestis*-positive carcasses were evaluated to identify delays. Test results were communicated from the laboratory through the UVRI epidemiology coordinator, and the recommendation to use IRS was made to district staff within a median of 1 day (range: 0–5) and mean of 1.1 days (95% CI: 0.3–1.9). District-level coordination to implement IRS initially added an additional 26.9 days on average to the total response time (95% CI: 8.7–45.2, range: 3–108, median: 7). Input from key stakeholders revealed that delayed mobilization of supplies, lack of funding for sprayer stipends, and poor motivation of IRS spray operators were, in part, the source of district-level delays to IRS intervention efforts. Key stakeholders from all levels of the program met to resolve these issues, and subcounty and district leadership resolved to provide timely reimbursement to IRS spray operators and to decentralize spray equipment. As a result, IRS response time decreased significantly after the meeting, from a median of 30 days to 7 days (Z = 3.08, *P* < 0.001) (Figure 4).

### Effectiveness of surveillance and response efforts.

In the years leading up to RFS, from 1999 to 2012, there were 59 laboratory-confirmed and 1,149 suspect and probable cases of human plague among the 83 villages participating in the RFS program. Other villages from Arua and Zombo districts with historical plague activity (*n* = ∼480) reported approximately 19 confirmed and 1,260 suspect and probable human plague cases during the same time period. During the surveillance period (July 1, 2013 to June 30, 2016), five laboratory-confirmed human cases of plague were reported from three villages outside the surveillance network, whereas no laboratory-confirmed plague cases were reported from the 83 participating villages. We chose not to compare case counts between villages inside and outside the network primarily because carcasses were accepted from all villages in the region regardless of how they learned about the program. Moreover, we responded to all positive carcasses and any human cases in the area regardless of their role in this study. Therefore, we did not have a valid comparison group to assess the impact of the program on plague case counts.

### Monthly call-based participant feedback.

UVRI staff attempted a total of 2,541 monthly phone calls to enrolled plague monitors to obtain feedback on the program and supplies. Of these calls, approximately half were received (*N* = 1,502). Single or multiple challenges were raised on 812 calls and included problems with program-critical supplies such as cell phones or solar chargers (*N* = 460) and accessory supplies, such as GPS units and batteries, insulated cold-storage boxes, or bicycles (*N* = 239), requests for disposable supplies, such as plastic bags, gloves, or insect repellent (*N* = 205), issues with the program administration or key stakeholders (*N* = 57), or other concerns (*N* = 33). Although cell phone coverage was available across the study area, service outages, and phone calls made to persons outside of service range were common, and resulted in 844 of the total 1,039 (81%) failed communication attempts.

## DISCUSSION

The RFS program described here is based on the premise that early recognition of plague epizootics coupled with IRS should reduce human plague case occurrence. During the first 3 years of this surveillance and response program in the plague-endemic West Nile region of Uganda, nearly 600 small mammal carcasses were submitted for testing. Carcass testing by DFA identified *Y. pestis* in 24 small mammals and led to IRS treatment and community sensitization of 17 villages in the region. No human plague cases were reported from participating villages during the evaluation period. Continued carcass submissions over 3 years by participating villages, as well as spontaneous submissions from neighboring or “out of network” villages demonstrated community engagement and support for the program. However, local, long-term sustainability of the program will require further evaluation of methods to reduce cost while maintaining the ability to recognize plague activity before the onset of human plague cases and provide adequate prevention resources.

IRS is intended to reduce flea loads on hut-dwelling rats and human contact with *Y. pestis*-infected fleas in the home, where most exposures in the West Nile region are presumed to occur.^5,6,12^ However, because IRS is not expected to reduce flea loads on plague-susceptible hosts away from the home, it is not anticipated to entirely disrupt the transmission of *Y. pestis* in enzootic and epizootic cycles away from the hut.^6,16^ Therefore, community sensitization was used to raise awareness among villagers of plague prevention strategies (e.g., avoiding handling sick or dead animals), recognition of plague symptoms, and the importance of seeking care early in the course of infection to improve outcomes of infection.^15^ Although we did not statistically evaluate whether the program significantly reduced human plague case occurrence, it is noteworthy that despite participating villages having a history of elevated case counts and evidence of plague activity in small mammals during the surveillance period, no human plague cases were reported from any of these villages. By contrast, five laboratory-confirmed human cases of plague were reported from three villages outside the surveillance network.

The absence of human plague cases after IRS and community sensitization are suggestive that a combined response to detection of rat falls is an effective plague prevention strategy. Although IRS is effective against on-host fleas, the costs of such interventions can be significant when supplies, training, and transportation are included.^12^ More cost-effective methods of flea control, such as topical insecticide rodent tubes^11^ may provide an alternate strategy. Given sufficient residual activity, this or other similar low-cost interventions could be deployed before the start of the plague season to reduce the risk of infectious flea bites or within the current program in response to rodent die offs. Alternately, community-level sensitization in response to reports of small mammal carcasses in the absence of IRS would be comparatively inexpensive and could reduce human plague cases and fatality rates through encouragement of plague prevention strategies and early care-seeking behavior. While the efficacy of this type of community awareness-only approach has not been evaluated for RFS, an ongoing plague detection and referral program in West Nile, may provide evidence that such a program can succeed.^23^

Natural fluctuations in the rodent and flea populations could likewise have contributed to the reduction in cases within participating villages, although given the proximity of these villages to those that reported plague cases, it seems unlikely that they would have experienced unique ecological shifts. It is difficult to draw conclusions, however because the scope of our evaluation did not include systematic live trapping efforts.

One limitation of this program was the use of DFA, a presumptive test for *Y. pestis*, to direct limited resources toward intervention efforts. Confirmation of DFA results can be achieved by culture isolation and specific bacteriophage lysis.^18^ However, the timely sourcing and shelf life of these specialized supplies proved problematic in this setting. Further, culture and phage lysis steps are time-consuming, and add days to the turnaround times for incoming samples.^24^ Therefore, for this program, DFA was chosen because the assay can detect the presence of *Y. pestis* in tissues of infected animals for days or weeks after death^24^ and because results can be obtained rapidly, to allow for rapid response when *Y. pestis* transmission is suspected. In the future, the integration of field-based diagnostic platforms, such as lateral flow (dipstick)^25^ or loop-mediated isothermal amplification assays,^26^ could improve sustainability of this type of program, given adequate sensitivity and specificity for field-collected carcass samples.

Although the RFS program was not structured to answer questions about IRS effectiveness and chemical residual activity against rodent-associated fleas, we nonetheless evaluated submission records to explore the idea that enzootic transmission was interrupted in villages after IRS treatment. We observed that no additional *Y. pestis*-positive carcasses were submitted from any of the 17 affected villages within the 2-month range of expected residual activity after IRS. However, 7 of the 17 village-based sprays were enacted late, from 22 to 113 days after the submission of a *Y. pestis*-positive carcass; in these villages, there were no *Y. pestis*-positive carcasses submitted during the interim between detection of the first positive carcass and IRS. Therefore, it is difficult to determine if the absence of additional *Y. pestis*-positive submissions from these villages was solely due to IRS, whether enzootic activity halted independently of the treatment, whether subsequent rodent deaths were occurring but were not detected by homeowners, or whether presumptive testing for *Y. pestis* from the carcass produced a false positive result. In future evaluations, control of host-associated fleas by the specific chemical to be applied by IRS could be measured and should be conducted in advance of scheduled changes to chemical types.

Rapid plague control interventions are important to halt enzootic transmission of *Y. pestis* and prevent human exposure to plague bacteria.^10^ Therefore, the timeliness of communications and response efforts were key indicators of program success. Rapid reporting, collection, submission, testing, and relaying of test result meant that at the conclusion of the third year of the program, village residents were informed of *Y. pestis* test results within 2 days of detecting small mammal deaths in their community. Communities experiencing small mammal deaths attributable to transmission of *Y. pestis* received timely information on the signs, symptoms, and treatment options for plague infection; throughout the duration of the 3-year period of evaluation, community sensitization efforts were consistently conducted within 4 days and never more than 9 days after the initial discovery of small mammal deaths. Issues with reimbursement of IRS technical staff and distribution of supplies initially slowed the deployment of village-based IRS; however, decisions made between key stakeholders during the first year of the program decreased response times significantly, from more than 40 days to fewer than 10. Overall, the time from a community member discovering a carcass to village-wide IRS decreased significantly over the 3 years of evaluation, meaning that affected communities were treated against infectious fleas sooner, thus reducing the likelihood of human cases.

*Rattus rattus*, the most common species submitted through the surveillance program, is highly susceptible to plague infection and maintains a close association with human habitations in the West Nile region.^8,27^ Because of this combination of characteristics, *R. rattus* in particular is a good sentinel for plague activity because increased mortality in and around huts may be more easily detected by village residents compared with mortality in sylvatic rodents and insectivores.^28^ Indeed, most small mammal carcasses reported through the program were found either indoors or in peridomestic areas. On the other hand, much of the mortality that village residents reported was not attributable to *Y. pestis* infection, resulting in a large number of samples tested yielding few plague-positive results.

To reduce the number and associated cost of reporting, retrieval, and testing of negative carcasses submitted through the RFS program, we explored limiting the definition of “rat fall” from the observation of one or more dead rodents, to include only the observation of more than one dead rodent in a single day because carcasses submitted in groups (> 1 per day) were more likely to test positive for *Y. pestis*. However, the harms associated with failure to apply appropriate interventions in the event of an active epizootic are high, and in the 3 years of evaluation, use of this adapted submission criteria would have resulted in treatment and sensitization of only 3 of the 17 (17.6%) affected villages. Another strategy to reduce program costs associated with testing a large number of negative samples might be to limit the number of months per year that carcasses can be submitted because most human cases in the region are reported during the months of September through December.^9^ However, during the 3 years of RFS evaluation, if we assumed a submission window of July through December to allow lead time in recognition of epizootics before human cases, only 15 of the 24 *Y. pestis*-positive carcasses (62.5%) would have been detected and would have resulted in failure to apply interventions 7 out of 17 times (41.2%). Therefore, using a limited time frame for submissions could not only introduce confusion over when to report carcasses, but could ultimately result in failure of the system to detect and respond to epizootic events when they occur outside a defined temporal window. Indeed, while most human cases in the region are reported from September through December, suspect human plague cases have been reported in every month of the year.^9^ Finally, because numbers of carcass submissions by month were highest during the “plague season,” testing on nearly 400 *Y. pestis*-negative carcasses would have been conducted during an annual July through December timeframe, reducing testing effort during the 3 years by only 204 samples (∼35%).

Other challenges to long-term sustainability of the program involve costs associated with material supplies provided to the VHTs. Participating VHTs reported problems with solar charging panels and cell phones, supplies that were critical to reporting rodent carcasses, in approximately 20% of monthly feedback calls. Personal use of these study supplies was encouraged, but reduced the longevity of these devices, and regular replacements were made at a cost to the program. The rapid expansion of mobile phone ownership throughout the sub-Saharan region and specifically in Uganda^29,30^ may diminish the need for program-supplied cell phones in the future. Furthermore, local participation in and acceptability of the surveillance program was high during the 3 years of evaluation, as evidenced by spontaneous reporting from “out of network” villages, repeated submissions from participating villages, and IRS acceptance rates. The potential expansion of cell-phone use in the area paired with continued local interest may support the long-term continuation of this early-warning plague surveillance program in West Nile.

In summary, implementation of a surveillance program for plague successfully identified rat falls, an early warning sign of local transmission, in 17 villages of the West Nile region of Uganda during 3 years of evaluation. Community engagement and participant input were key for making improvements to the program so that in the final year of evaluation, IRS and community sensitization were initiated within 10 days of notification. Among the villages where plague activity was detected and IRS was conducted, within 2 months of IRS application, no additional *Y. pestis*-positive carcasses were submitted, and no human cases were reported. Given these successes, we consider this RFS program to be a useful means of plague prevention in the West Nile region of Uganda, where plague is endemic but difficult to predict using broad-scale geographic and climate indicators.
